# Post-operative outcomes and complications of suspensory loop fixation device versus hook plate in acute unstable acromioclavicular joint dislocation: a systematic review and meta-analysis

**DOI:** 10.1007/s10195-017-0451-1

**Published:** 2017-02-25

**Authors:** Alisara Arirachakaran, Manusak Boonard, Peerapong Piyapittayanun, Wichan Kanchanatawan, Kornkit Chaijenkij, Akom Prommahachai, Jatupon Kongtharvonskul

**Affiliations:** 1 Orthopedics Department, Police General Hospital, Bangkok, Thailand; 2 Orthopedics Department, Srinakarin Hospital, Khonkan, Thailand; 3 Orthopedic Department, Lerdsin General Hospital, Bangkok, Thailand; 4 Orthopedics Department, College of Sports Science and Technology, Mahidol University, Bangkok, Thailand; 5 Orthopedic Department, Aek Udon International Hospital, Udontani, Thailand; 6 Sport and orthopedic center, Samitivej Hospital, Bangkok, Thailand

**Keywords:** Hook plate, Loop suspensory fixation, AC injury, Systematic review, Tightrope, Endobutton

## Abstract

**Background:**

Treatment of acute (≤3 weeks) acromioclavicular joint dislocation type III–VI is still controversial. Currently, the two modern techniques that are widely used are hook plate (HP) fixation and coracoclavicular ligament fixation using a suspensory loop device that consists of either a tightrope (single or double), endo-button (single or double), or synthetic ligament and absorbable polydioxansulfate sling.

**Materials and methods:**

This systematic review was conducted according to the PRISMA guidelines. Relevant studies that reported Constant-Murley score (CMS), Pain Visual Analog score (VAS) and postoperative complications of either technique were identified from Medline and Scopus from inception to 5 October 2015.

**Results:**

Sixteen studies were included for the analysis of HP fixation, and 25 studies were included for analysis of loop suspensory fixation (LSF). Pooling of mean CMS and VAS scores gave 90.35 (95% CI 87.16, 93.54), 1.51 (95% CI 0.73, 2) in the HP group, and 92.48 (95% CI 90.91, 94.05), 0.32 (95% CI 0, 0.64) in the suspensory loop devices group, respectively. The pooled unstandardized mean differences (UMD) scores of CMS and VAS in LSF were 2.13 (95% CI −1.43, 5.69) and −1.19 (95% CI −2.03, −0.35) when compared to hook plating. The pooled prevalence of LSF and hook plating were 0.08 (95% CI 0.06, 0.10) and 0.05 (95% CI 0.02, 0.08) scores. The chance of having complications in the LSF group was 1.69 (95% CI 1.07, 2.60), which was statistically significantly higher than in the HP group.

**Conclusion:**

LSF have higher shoulder function scores (CMS) and lower postoperative pain when compared to HP fixation; however, there are higher complication rates with LSF when compared to hook plating.

**Level of evidence:**

IV.

## Introduction

Acromioclavicular (AC) joint (ACJ) dislocation is a common injury in active young adults [5]. The prevalence was approximately 9% of shoulder girdle injuries [3, 28]. AC dislocation is associated with AC and coracoclavicular (CC) ligaments injuries [37]; such injuries are classified into type I–VI on the basis of the radiographic findings using the Rockwood criteria [26, 37]. Non-operative treatment has generally been accepted as the gold standard of treatment in Rockwood I and II lesions [52], whereas the optimal method of treatment for grade III–VI lesions remains a matter of controversy [16, 30, 59]. Although type IV–VI injuries are treated operatively because of their severe instability [45], treatment for type III injury is still controversial [22, 52]. The aim of any surgical approach addressing the instability of the ACJ should be an anatomic reduction and restoration of normal arthrokinetics [18]. Surgical methods employed for the treatment of AC dislocations include extraarticular fixation by CC restoration with metallic cables, autologous ligaments or LARS artificial ligaments, transarticular fixation by hook plate (HP) and Kirschner wires. Kirschner wires and threaded pins are frequently used for temporary fixation of the ACJ. However, serious concerns still exist regarding pin migration or breakage, pin-site infection, fixation failure, and recurrent instability after pin removal [14, 21, 31]. Currently, two modern techniques that are widely used include HP fixation [2, 7, 10, 29, 35, 59] and CC ligament fixation using a suspensory loop device [tightrope (single or double), endo-button (single or double), synthetic ligament and absorbable polydioxansulfate sling (PDS)] [7, 8, 10, 12, 19, 31, 32, 35, 36, 39, 51]. Many case series have reported safe and effective results with the treatment of acute unstable ACJ dislocations by loop suspensory fixation (LSF) [1, 8, 9, 12, 20, 24, 40, 42, 43, 50, 55] and hook plating [7, 17, 25, 38, 39, 49, 56, 57]. More recently, several retrospective cohort studies have aimed to investigate the results of treatment for unstable acute AC dislocations (type III–VI) with HP and LSF. However, there has been no consensus as to which is better for treatment of acute unstable ACJ dislocation. Some studies show benefits of the AC or CC augmentations in pain and Constant-Murley score (CMS) [29, 59], whereas other studies do not [2, 10, 18]. However, LSF consists of variable types of fixation, which include tightrope (single or double), endo-button (single or double), synthetic ligaments and absorbable PDS. Moreover, no high quality methodological quality study [prospective cohort or randomized controlled trials (RCT)] has recently been published. We hypothesized that the impact of each type of fixation with LSF would be comparable to that the HP fixation in acute unstable AC joint dislocation. We therefore conducted a systematic review and meta-analysis that analyzes the available literature, with the aim of comparing the outcomes and safety of LSF, including all different types of implants (tightrope, endo-button, synthetic ligament and PDS) with HP fixation for treatment of acute unstable AC joint dislocation. These clinical outcomes consist of the CMS, Pain Visual Analog score (VAS) and postoperative complications.

## Materials and methods

### Search strategy

The Medline and Scopus databases were used for identifying relevant studies published in English since the date of inception to 5 October 2015. The PubMed and Scopus search engines were used to locate studies with the following search terms: {[(acromioclavicular joint) OR AC joint] AND [(separation) OR dislocation OR trauma OR injury] AND [(hook plate) OR locking plate OR fixation OR tightrope OR dogbone] AND [(Constant score) OR Constant Murley scale OR CMS OR pain OR UCLA]}. Search strategies for Medline and Scopus are described in detail in the “Appendix 1”. References from the reference lists of included trials and previous systematic reviews were also explored.

### Inclusion criteria

Observational studies (e.g., cross-sectional or cohort) that reported clinical outcomes of hook plate or fixation of the CC ligament using an LSF device for treatment of acute unstable ACJ injury were eligible if they met the following criteria:

- Reported at least one of the following outcomes: CMS, VAS, and postoperative complications.
- Had sufficient data to extract and pool, i.e., the reported mean, standard deviation (SD), number of subjects according to treatments for continuous outcomes, and number of patients according to treatment for dichotomous outcomes.

The use of a combination of LSF or HP with other methods of fixation and non-English studies were excluded. The reference lists of the retrieved articles were also reviewed to identify publications on the same topic. Where there were multiple publications from the same study group on the same population, the most complete and recent results were used.

### Data extraction

Two reviewers (J.K. and A.A.) independently performed data extraction using standardized data extraction forms. General characteristics of the study [i.e., mean age, gender, body mass index (BMI), mean follow up time, mean duration after injury, pain VAS and CMS score at baseline] were extracted. The number of subjects, mean, and SD of continuous outcomes (i.e., VAS and CMS) between groups were extracted. Cross-tabulated frequencies between treatment and all dichotomous outcomes (post-operative complications) were also extracted. Any disagreements were resolved by discussion and consensus with a third party (M.B.).

### Outcomes of interest

The outcomes of interests included CMS, VAS, and postoperative complications. These outcomes were measured as reported in the original studies, which were VAS pain scale from 0 to 10 cm (lower values of these scores refer to better outcomes), CMS (0–100, higher values are equivalent to better outcomes). Postoperative complications (wound problems, loss of reduction, implant migration and osteolysis) were considered.

### Statistical analysis

For continuous outcomes (CMS and VAS), unstandardized mean differences (UMD) was pooled and calculated using the method as follows [53]: UMD $(d_{I} ) = \bar{x}_{1i} - \bar{x}_{2i} ,\,\text{var} (d_{I} ) = \frac{{{\text{sd}}_{1i}^{2} }}{{n_{1i} }} + \frac{{{\text{sd}}_{2i}^{2} }}{{n_{2i} }},\,w_{I} = \frac{1}{{\text{var} (d_{I} )}},$ where *w*_I_ is the weighting factor, *d*_I_ is the standardized/unstandardized difference of means, *D*_I_ is the pooled difference of means, *n*_1i_ and *n*_2i_ are the number of subjects in group 1 and 2, *n*_I_ is *n*_1i_ + *n*_2i_, sd_I_ is the pooled SD, var(*d*_*I*_) is variance of difference, and the subscript *I* is the study *I*. Heterogeneity was checked using *Q* statistic as follows: $Q = \mathop \sum \nolimits_{i}^{k} w_{i} (d_{i} - D)^{2} ,\;D = \frac{{\mathop \sum \nolimits_{i = 1}^{k} w_{i} d_{i} }}{{\mathop \sum \nolimits_{i = 1}^{k} w_{i} }},\;w_{i} = \frac{1}{{\text{var} (d_{i} )}}.$ The *Q* statistic follows a Chi square distribution with *k* − 1 degrees of freedom (*df*).

For dichotomous outcomes (complications), the prevalence was pooled and calculated using the inverse variance method as follows [53] $\bar{p} = \frac{{\sum w_{i} p_{i} }}{{\sum w_{i} }}$ where *p* was the pooled prevalence, *p*_i_ was the prevalence of complications of each study,* w*_i_ was 1/var(*p*_i_), which was the weight of each study. Heterogeneity of prevalence across studies *p* was checked as follows: $\sum w_{i} (p_{i} - \bar{p} )^{2}$. The *Q* statistic follows a $\chi^{2}$ distribution with number of studies (*k*) − 1 degree of freedom (*df*). The degree of heterogeneity was also quantified using the *I* ^2^ statistic [15]. This value can range from 0 to 100%, the closer to 100%, the higher the heterogeneity. If heterogeneity was present, between studies variation was then estimated as follows: $\tau^{2} = \frac{Q - (k - 1)}{{\sum w_{i} - \frac{{\sum w_{1}^{2} }}{{\sum w_{1} }}}}$ if *Q k* 1 or 0 otherwise. This was used to calculate a weight term that accounted for variations between studies $w_{i}^{*} = \frac{1}{{\text{var} (p_{1} ) = \tau^{2} }}$ and then the pooled prevalence was estimated using the random effects model as follows: $95\% \;{\text{CI}} = \bar{p} ^{*} \pm \frac{1.96}{{\sqrt {\sum w_{i}^{*} } }}.$

Meta-regression analysis was then applied to explore causes of heterogeneity [15, 54]. Coverable parameters, i.e., type of implants (single and double loops), mean age, percentage male, and type of injuries (III, IV, V and VI) were considered in the meta-regression model. Power of the test for meta-regression was also assessed [44]. The UMD and odds ratio (OR) were estimated by indirect meta-analysis using a random effects model, otherwise a fixed effects model was applied. All analyses were performed using STATA version 14.0 [48].

## Results

**Fig. 1:**
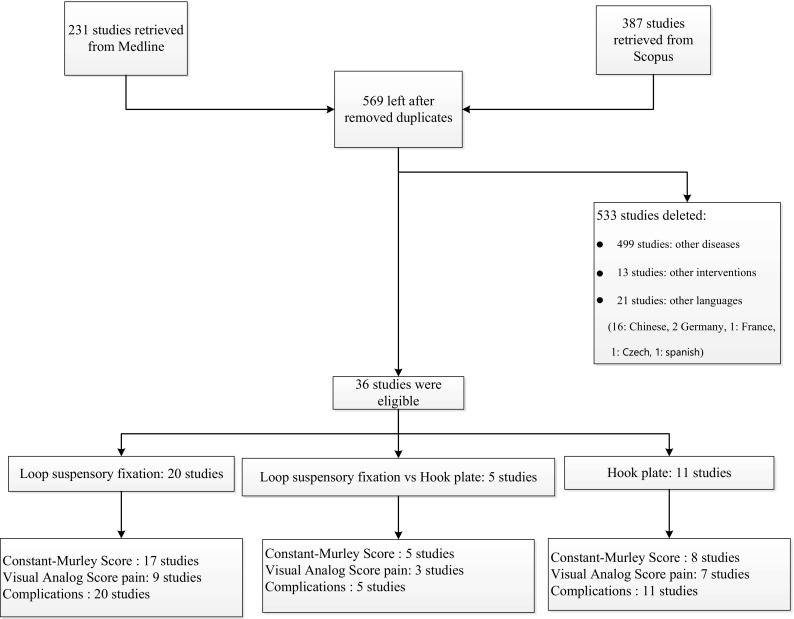
Flow of study selection

In all, 231 and 387 studies were identified from Medline and Scopus respectively, as described in Fig. 1, of which 49 studies were duplicates, leaving 569 studies for review of titles and abstracts. Of these, 36 articles were relevant and the full papers were retrieved. Characteristics of these studies are described in Table 1: 28 studies were case series reports, 7 were cohort studies and 1 study was a cross-sectional study. Twenty studies reported results of LSF, 11 studies reported results of HP fixation, and 5 studies compared LSF to HP fixation. All 36 studies reported postoperative complications, 25 studies for LSF (22 studies were reported CMS and 12 studies were VAS for pain), and 16 studies for HP fixation (13 studies were reported CMS and 10 studies were VAS for pain). Mean age, percentages of male gender, duration from injury and mean follow up of LSF participants varied from 26 years to 45.6 years, 72.7% to 94.4%, 4.2 days to 13 days and 3 months to 70 months, while HP varied from 29 years to 42.3 years, 84.4% to 100%, 3.5 days to 9.2 days and 3 months to 50.4 months. In all 36 studies, fixation was performed in ACJ injury types III–VI. Twenty-two studies were type III and V, 6 studies were type III, 6 studies were type V, 1 study was type III–IV, 1 study was type IV–V and 1 study was type IV–VI. In the LSF group, 13/24 studies used arthroscopically assisted techniques, as did 4/16 studies in the hook plate group; 12/25 studies used double loop and 13/25 studies used single loop fixation. In the HP group, 14/16 studies reported the time of plate removal, with 8 studies removing the plates within 3 months of initial operation, 4 studies removing the plates at 4 months, and 2 studies removing the plates after 4 months.

**Table 1:** Characteristics of included studies

Author	Year	Type of study	Follow up (months)	Arthroscopic assisted	Suspensory loop (single or double) or HP	Implant types	Implant removal time (month)	Age	Type of AC injury	Male (%)	Time interval from injury to surgery (days)	Outcome
Stam L	1991	Case series	46.8	N	Single	Dacron	–	40	III–V	73.9	4.4	Complications
Dimakopoulos P	2006	Case series	33.2	N	Double	Ethibond	–	33.5	III/V	91.2	10	CMS, complications
Ryhanen J	2006	Case series	12	N	Single	C-hook	3	37	III	–	–	Complications
Choi S	2008	Case series	41.2	N	Single	Ethibond	–	33.5	III–V	80	–	VAS, complications
Murena L	2009	Case series	31	N	Double	Endobutton	–	33.3	III–V	93.8	4.3	CMS, complications
Greiner S	2009	Case series	70	N	Single	PDS	–	35.3	III–V	86	–	CMS, complications
Salzmann GM	2010	Case series	24	Y	Double	Tightrope	–	37.5	III–V	91.3	11.3	CMS, VAS, complications
Scheibel M	2011	Case series	26.5	Y	Double	Tightrope	–	38.6	III/V	92.9	7.3	CMS, complications
Wai HF	2011	Case series	12	N	Double	Endobutton	–	42.8	III–V	86.7	6.5	CMS, VAS, complications
El Sallakh SA	2012	Case series	24	Y	Single	Tightrope	–	30	V	90	–	CMS, complications
Sandmann GH	2012	Case series	32	N	Double	Tightrope	–	39	III–V	91	5	CMS, VAS, complications
Beris A	2013	Case series	18.25	N	Single	Tightrope	–	27.5	III–IV	75	5	CMS, VAS, complications
Kraus N	2013	Cohort	24	N	Double (V)	Tightrope	–	37.7	V	93.3	–	CMS, complications
Kraus N	2013	Cohort	24	N	Double	Tightrope	–	40.9	V	92.3	–	CMS, complications
Venjakob AJ	2013	Case series	58	Y	Double	Tightrope	–	–	III–V	91.3	–	CMS, VAS, complications
Spolitil M	2014	Case series	5	Y	Single	Tightrope	–	33	III–V	84.2	–	CMS, VAS, complications
Acar MA	2015	Case series	13.6	Y	Single	Ziploop	–	43.4	III	92.3	7.92	CMS, VAS, complications
Katsenis DL	2015	Case series	42	N	Single	Flipptack	–	35.5	IV–V	76	4.2	CMS, complications
Pan Z	2015	Case series	24	Y	Single	Endobutton	–	26	III/V	72.7	6.1	CMS, complications
Shin SJ	2015	Case series	25.6	Y	Single	Tightrope	–	45.4	III–V	94.4	6.1	CMS, complications
Struhl S	2015	Case series	62.4	Y	Single	Endobutton	–	45.7	III–V	88.9	13	CMS, complications
Koukakis A	2008	Case series	10.6	N	HP	HP	2–3	–	III–V	–	7.3	CMS, VAS, complications
Salem KH	2009	Case series	30	N	HP	HP	2.5	41	III–V	92	7	CMS, complications
Kienast B	2011	Case series	36	N	HP	HP	3	38.4	III–V	83.7	6	Complications
Francesco AD	2012	Case series	12	N	HP	HP	3	27.5	III/V	66.7	3.5	CMS, complications
Gille J	2013	Case series	7	Y	HP	HP	–	–	III/V	–	–	CMS, complications
Sarrafan N	2012	Case series	12	N	HP	HP	8	38	III	90	–	VAS, complications
Guizzi P	2012	Case series	21	N	HP	HP	–	–	III	–	–	CMS, complications
Heideken JV	2013	Case control	32	N	HP	HP	4	40	V	77.3	0.3	CMS, VAS, complications
Jafary D	2014	Case series	19	N	HP	HP	5	40.3	III/V	91.7	–	CMS, complications
Steinbacher G	2014	Case series	50.4	N	HP	HP	4	29	III	73.7	–	VAS, complications
Kumar N	2015	Case series	23.5	N	HP	HP	4	34.24	III	100	9.06	CMS, complications
Escher A	2011	Cohort	31.2	N	Single vs HP	PDS	4	42.3	V	88.5	8.5	VAS, CMS, complications
Andreani L	2014	Cohort	3	N	Single vs HP	Tightrope	3	–	IV–VI	100	7.2	CMS, complications
Jensen G	2014	Cohort	48	Y	Double vs HP	Tightrope	3	39	III/V	91.1	–	VAS, CMS, complications
Metzlaff S	2016	Cohort	32	Y	Single vs HP	FlippTack	3	37.6	III/V	79.5	14	CMS, complications
Yoon JP	2015	Cohort	–	Y	Double vs HP	LIGASTIC	2	40	V	78.6	9.2	VAS, CMS, complications

* HP* Hook plate,* AC* acromioclavicular, *PDS* polydioxansulfate sling, *CMS* Constant-Murley score, *VAS* Visual Analog score

### Pooled mean CMS in LSF and HP

Thirty-eight studies using LSF and HP fixation in high-grade acute ACJ injury were included for pooling of means and 95% confidence intervals (CI) (Table 2). Among 23 LSF studies, 15 were single bundle fixation, 10 were double bundle fixation, 12 were arthroscopically assisted and 13 were open technique. In terms of CMS, with the LSF group containing 663 patients and HP fixation having 394 patients, the pooled mean CMS of LSF varied greatly across studies (*I*_2_ = 75.2) and was 92.48 (95% CI 90.91, 94.05) (Table 2). The pooled mean of CMS of 16 HP studies varied across studies (*I*_2_ = 85.47) at 90.35 (95% CI 87.16, 93.54). From the result of the indirect meta-analysis, the pooled UMD were 2.13 (95% CI −1.43, 5.69), which translates to the mean CMS of LSF scoring 2.13 higher than HP fixation but the difference was not statistically significant.

**Table 2:** Estimation of the pooled mean of CMS and VAS pain of loop suspensory fixation (LSF) and HP

Author	Follow up (months)	Age (years)	Male (%)	*N*	CMS		VAS	
					Mean	SD	Mean	SD
Dimakopoulos P	33.2	33.5	91.2	15	93.5	8.05	–	–
Choi S	41.2	33.5	80	26	89.5	8.13	–	–
Murena L	31	33.3	93.8	16	97	5.48	–	–
Greiner S	70	35.3	86	50	91.7	8.7	–	–
Salzmann GM	24	37.5	91.3	23	94.3	3.2	0.25	0.5
Scheibel M	26.5	38.6	92.9	37	91.75	7.51	–	–
Wai HF	12	42.8	86.7	15	89.15	7.61	0.2	0.62
El Sallakh SA	24	30	90	10	96.4	1.44	–	–
Sandmann GH	32	39	91	33	94.3	7.1	0.5	0.6
Beris A	18.25	27.5	75	12	94.7	6.3	0.17	0.58
Kranus N	24	37.7	93.3	15	88.5	1.85	–	–
Kranus N	24	40.9	92.3	13	92.2	4.62	–	–
Venjakob AJ	58	–	91.3	23	91.5	4.7	0.32	0.6
Spolitil M	5	33	84.2	19	89.7	10.9	2.11	1.76
Acar MA	13.6	43.4	92.3	13	84.46	5.5	0.69	1.3
Katsenis DL	42	35.5	76	50	93.02	4.63	–	–
Pan Z	24	26	72.7	22	92.51	2.4	–	–
Shin SJ	25.6	45.4	94.4	18	97	2.19	–	–
Struhl S	62.4	45.7	88.9	9	98.8	1.1	–	–
Escher A	31.2	42.3	88.5	52	94.6	1	0.8	0.2
Andreani L	3	–	100	28	90	7.5	–	–
Jensen G	48	39	91.1	56	82.5	14.36	1.3	1.7
Metzlaff S	32	37.6	79.5	44	93.6	3.4	–	–
Yoon JP	–	40	78.6	42	89.2	3.5	1.3	1.3
Pooled mean LSF (95% CI)					92.48 (90.91, 94.05)		0.32 (0, 0.64)	
Koukakis A	10.6	–	–	16	96.4	6.7	0.87	1.76
Salem KH	30	41	92	23	97	1.94	–	–
Francesco AD	12	27.5	66.7	42	91.79	9.2	–	–
Sarrafan N	12	38	90	30	–	–	4	1.73
Escher A	31.2	42.3	88.5	52	91.2	2.2	0.77	0.2
Guizzi P	21	–	–	12	93.23	6.47	–	–
Gille J	7	–	–	3	90.75	5.2	–	–
Heideken JV	32	40	77.3	19	90	1.76	2.5	1.76
Andreani L	3	–	100	28	75	5.8	–	–
Jensen G	48	39	91.1	56	73.8	24.24	1.7	2.3
Metzlaff S	32	37.6	79.5	44	92.8	3.8	–	–
Steinbacher G	50.4	29	73.7	19	–	–	1.8	0.58
Jafary D	19	40.3	91.7	24	94.5	8.77	–	–
Kumar N	23.5	34.24	100	33	91.8	13.07	–	–
Yoon JP	–	40	78.6	42	90.2	9.9	1.6	1.5
Pooled mean HP (95% CI)					90.35 (87.16, 93.54)		1.51 (0.73, 2.00)	
UMD (95% CI) of LSF vs hook plate					2.13 (−1.43, 5.69)		−1.19 (−2.03, −0.35)	

* UMD* Unstandardized mean differences

### Pooled mean VAS in LSF and HP

Ten LSF studies and seven hook plate studies were pooled for VAS pain scores. For the LSF group of 288 patients and HP group of 234 patients, the pooled mean VAS of LSF and HP were homogenous across studies (*I* ^2^ = 0 and 15.09), scoring 0.32 (95% CI 0, 0.64) and 1.51 (95% CI 0.73, 2) (Table 2). From indirect meta-analysis, the pooled UMD were −1.19 (95% CI −2.03, −0.35), translating to the mean VAS of LSF being about 1.2 scores statistically significantly lower when compared to hook plating.

### Pooled prevalence of LSF and HP

Twenty-five LSF studies and 16 hook plate studies pooled the prevalence of complications after fixation. For the LSF group of 701 patients and HP group of 668 patients, the pooled prevalence of LSF and HP had mild to moderate degrees of heterogeneity across studies (*I* ^2^ = 24.27 and 42.13), with scores of 0.08 (95% CI 0.06, 0.10) and 0.05 (95% CI 0.02, 0.08) (Table 3). From indirect meta-analysis, the difference in the risk of having complications between two groups was 1.69 (95% CI 1.07, 2.60), indicating that the chance of having a wound problems, loss of reduction, implant migration and osteolysis in the LSF group was about 1.7 times statistically significant higher than in the HP group (Table 3).

**Table 3:** Estimation of the pooled prevalence of post-operative complication of LSF and HP

Author	Follow up (months)	Age (years)	Male (%)	*N*	Complication	
					Yes	No
Stam L	46.8	40	73.9	23	0	23
Dimakopoulos P	33.2	33.5	91.2	15	2	32
Ryhanen J	12	37	–	15	1	14
Choi S	41.2	33.5	80	26	1	19
Murena L	31	33.3	93.8	16	2	14
Greiner S	70	35.3	86	50	4	46
Salzmann GM	24	37.5	91.3	23	1	22
Scheibel M	26.5	38.6	92.9	37	0	40
Wai HF	12	42.8	86.7	15	2	13
El Sallakh SA	24	30	90	10	0	10
Sandmann GH	32	39	91	33	3	30
Beris A	18.25	27.5	75	12	0	12
Kranus N	24	37.7	93.3	15	0	13
Kranus N	24	40.9	92.3	13	1	14
Venjakob AJ	58	–	91.3	23	2	21
Spolitil M	5	33	84.2	19	3	16
Acar MA	13.6	43.4	92.3	13	1	12
Katsenis DL	42	35.5	76	50	6	50
Pan Z	24	26	72.7	22	2	20
Shin SJ	25.6	45.4	94.4	18	8	10
Struhl S	62.4	45.7	88.9	9	4	5
Escher A	31.2	42.3	88.5	52	0	25
Andreani L	3	–	100	28	2	17
Jensen G	48	39	91.1	56	12	14
Metzlaff S	32	37.6	79.5	44	1	23
Yoon JP	–	40	78.6	42	6	12
Pooled prevalence LSF (95% CI)				0.08 (0.06, 0.10)		
Koukakis A	10.6	–	–	16	0	16
Salem KH	30	41	92	23	7	16
Kienast B	36	38.4	–	225	24	201
Francesco AD	12	27.5	66.7	42	5	37
Sarrafan N	12	38	90	30	1	29
Escher A	31.2	42.3	88.5	52	5	22
Guizzi P	21	–	–	12	1	1
Gille J	7	–	–	3	0	3
Heideken JV	32	40	77.3	19	0	19
Andreani L	3	–	100	28	0	7
Jensen G	48	39	91.1	56	19	11
Metzlaff S	32	37.6	79.5	44	0	20
Steinbacher G	50.4	29	73.7	19	0	19
Jafary D	19	40.3	91.7	24	2	22
Kumar N	23.5	34.24	100	33	0	33
Yoon JP	–	40	78.6	42	9	15
Pooled prevalence HP (95% CI)				0.05 (0.02, 0.08)		
RR of LSF vs HP				1.69 (1.07, 2.60)		

### Sources of heterogeneity

Meta-regression was applied to explore the cause of heterogeneity by fitting a co-variable (i.e., age, percentage of sex, type of AC injury, approach, number of bundle, time of plate removal, duration before surgery and type of studies level), and meta-regression was applied to assess this. None of the co-variables could explain the heterogeneity. However, the type of approaches and number of bundles of fixation might be the source of heterogeneity. Therefore, subgroup analyses were performed as described in Table 4.

**Table 4:** Estimation of the pooled mean CMS, VAS, and prevalence of complications in the LSF subgroup

Subgroup analysis	Mean	95% CI	*Q* test	*df*	*I*_2_	*P* value
CMS						
Pooled mean CMS of arthroscopic assisted LSF	92.41	90.68, 94.13	21.64	10	49.17	0.027
Pooled mean CMS of open LSF	92.40	89.89, 94.92	65.45	11	83.19	<0.001
Arthroscope versus open LSF	0.01 (−3.04, 3.06)					
Pooled mean CMS of single bundle LSF	93.31	91.30, 95.32	51.32	12	76.62	<0.001
Pooled mean CMS of double bundle LSF	91.48	89.49, 93.48	22.32	10	55.19	0.016
Single versus double LSF	1.83 (−1.22, 4.88)					
Pooled mean CMS of HP within 3 months	88.42	82.75, 94.10	407.28	6	98.53	<0.001
Pooled mean CMS of hook plate after 3 months	91.08	89.85, 92.32	10.08	3	70.23	0.018
Plate remove within versus after 3 months	−2.66 (−14.05, 8.73)					
Pooled mean CMS of HP open	90.08	86.66, 93.50	82.02	11	86.59	<0.001
VAS						
Pooled mean VAS of arthroscopic assisted LSF	1.14	0.07, 1.49	2.25	5	0	0.813
Pooled mean VAS of open LSF	0.66	0.08, 1.16	0.76	3	0	0.858
Arthroscope versus open	0.48 (−0.48, 1.44)					
Pooled mean VAS of single bundle LSF	1.13	0.06, 1.44	1.65	3	0	0.649
Pooled mean VAS of double bundle LSF	0.41	0.21, 1,.15	1.17	5	0	0.948
Single versus double	0.72 (−0.69, 2.13)					
Pooled mean VAS of HP within 3 months	1.50	1.19, 1.86	2.65	2	24.37	0.267
Pooled mean VAS of HP after 3 months	2.23	1.11, 3.35	175.40	3	98.3	<0.001
Plate remove within versus after 3 months	−0.73 (−3.06, 1.60)					
Pooled mean VAS of HP open	1.51	0.75, 2.27	7.07	6	15.09	0.315

Complications	Prevalence	95% CI	*Q* test	*df*	*I*_2_	*P* value
Pooled prevalence of arthroscopic assisted LSF	0.1	0.04, 0.15	29.99	11	63.32	0.002
Pooled prevalence of open LSF	0.08	0.05, 0.11	3.02	13	0	0.998
Arthroscope versus open LSF	1.31 (0.77, 2.23)					
Pooled prevalence of single bundle LSF	0.07	0.04, 0.09	22.82	14	38.65	0.063
Pooled prevalence of double bundle LSF	0.1	0.06, 0.13	8.33	10	0	0.597
Single versus double	0.67 (0.38, 1.15)					

## Discussion

From the current available evidence, this systematic review and meta-analysis has shown the following: LSF implants have higher shoulder function and lower shoulder pain reported by CMS and VAS scores of 2.2 and 1.2 points, respectively, when compared to HP fixation. However, LSF displayed a higher complication rate after surgery, (wound problems, loss of reduction, implant migration and osteolysis) being 1.7 times higher than HP fixation in acute unstable ACJ injury.

Of the previously published studies [2, 10, 18, 29, 59], there have been no high quality studies comparing the results of these two fixation methods. Although there are comparative studies reporting results between the two groups, there is still no clear consensus as to which is preferable. From this study, we have additional evidence that LSF displays a higher improvement of CMS and VAS scores when compared to HP fixation. However, LSF has a higher risk of postoperative complications when compared to HP fixation. The mean CMS, VAS Pain score and prevalence of complications among included studies was heterogeneous, possibly due to methodological and clinical differences. Attempts were made to explore sources of heterogeneity by considering clinical (i.e., age, percentage of sex, type of AC injury, approach, number of bundle, time of plate removal and duration before surgery) and methodological variables (i.e., type of study) in the meta-regression model. None of the co-variables could explain the heterogeneity. However, the degree of heterogeneity did not decrease after pooling all subgroups, indicating the presence of other sources of heterogeneity. There are several important clinical factors that may have had an effect on the results, including the use of two different approaches (arthroscopic or open) and two different implant designs (single or double) that are suspected to be the source of heterogeneity of the LSF devices. Although LSF shows higher complication rates postoperatively, HP fixation is a double procedure that also requires a second surgery for plate removal. After subgroup analyses, the results show that there are still no statistically significant differences in CMS, VAS and complications between different approaches and the number of bundle fixation (Table 4). The timing of the second operation for plate removal displays no statistically significant difference for CMS and VAS outcomes comparing before 3 months and after 3 months of the initial surgery.

Although we were unable to find the source of heterogeneity by meta-regression and subgroup analysis (two different approaches, two different implant designs of LSF devices and the plate removal time after hook plating), several factors must be considered in clinical implementation. Firstly, there is either single [1, 2, 4, 6, 9, 10, 12, 20, 29, 34, 38, 43, 46, 47, 50] or double (V anatomic shape [24] with parallel [8, 18, 33, 40, 42, 55, 58, 59]) bundle LSF fixation, in which double bundles demonstrate higher function scores (CMS) and lower pain VAS. Within the double bundle groups, no significant differences regarding clinical or radiologic results have been found [24]. Secondly, arthroscopic techniques have recently been described in the treatment of acute AC dislocation [1, 9, 18, 29, 34, 40, 42, 43, 46, 50, 55, 59]. With the use of an arthroscopic approach, LSF seems to have higher pain VAS when compared to open LSF [2, 4, 6, 8, 10, 12, 20, 24, 33, 38, 47, 58], but shows no differences in terms of function, outcomes or complication rates. For HP fixation, most studies used an open approach [7, 13, 17, 21, 23, 39, 41, 49, 56], with only one study using an arthroscopic approach [11]. However, after subgroup analysis, there were no differences in pain function and complication outcomes. As for the time of implant removal after initial surgery in the hook plating group, pain VAS was lower when the plate was removed within 3 months [2, 7, 18, 21, 23, 29, 39, 59], while the CMS function score was higher when the plate was removed after 3 months [10, 17, 25, 41, 49, 56] (Table 4). Therefore, the recommended time to remove the implant will depend on the symptoms of each individual patient. If there is persistent pain after surgery, the HP should be removed before 3 months. If the patient has no pain but limited shoulder function; the implant should not be removed prior to 3 months postoperatively.

The strength of this study is that it has a high power to detect a clinical difference between two implant fixations (the minimal clinically important difference of VAS is 1.2 points), with a power of 100% to detect this margin, and a type I error of 1%. This study uses adequate methodology of systematic review in accordance with* Preferred Reporting Items for Systematic Reviews and Meta-Analyses* (PRISMA) guidelines [27], as well as providing exploration and reduction of the heterogeneity of the studies using subgroup analysis and adequate statistical analysis.

There were some limitations in this study. Firstly, the quality of studies for the meta-analysis was not high. Ideal evidence for systematic review is an RCT, which is most commonly used in testing the efficacy of surgery. Only 5 trials included were comparative studies (retrospective cohort studies) while 31 trials included were case series reports. This could be a possible source of bias between groups due to the opportunity for selection and different baseline characteristics. Secondly, heterogeneity remains an important factor to be considered in the conduct and interpretation of meta-analysis, and the heterogeneity between studies was great. We applied the random effects meta-analysis to adjust for the differences between studies, and the possible causes of heterogeneity were explored if covariate data at baseline (e.g., age, percentage of sex, type of AC injury, approach, number of bundle, timing of plate removal, duration before surgery and type of studies) were available. The third limitation is that indirect meta-analysis was used for calculating the mean difference and odds ratio between the two groups, due to the fact that most included studies were case series reports of only one technique. The fourth limitation is that there are other outcomes of interest that can be used to compare LSF and HP fixation such as operation cost or post-operative satisfaction and quality of life. However, these factors could not be analyzed because of insufficient data. The last limitation is that most studies had a mean follow up time of approximately 1–2 years; therefore mid-term to long-term effects of the different types of fixation are still unknown.

In summary, for acute high-grade ACJ injuries, both HP and LSF had acceptable post-operative outcomes. LSF provided better postoperative shoulder function (CMS) when compared to HP fixation, but the difference was not statistically significant. LSF provided clinically and statistically significant lower pain VAS when compared to HP fixation. However, LSF had higher complication rates when compared to the HP fixation group. This study shows that both options have advantages and disadvantages and should be chosen based on patient status. In the future, prospective randomized controlled studies are needed to confirm these findings as the current literature is still insufficient.

## Appendix

### Search term and strategy

#1 acromioclavicular joint

#2 AC joint

#3 #1 OR #2

#4 separation

#5 dislocation

#6 trauma

#7 injury

#8 #4 OR #5 OR #6 OR #7

#9 hook plate

#10 locking plate

#11 fixation

#12 tightrope

#13 dogbone

#14 #9 OR #10 OR #11 OR #12 OR #13

#15 Constant score

#16 Constant Murley scale

#17 CMS

#18 pain

#19 UCLA

#20 #15 OR #16 OR #17 OR #18 OR #19

#21 #3 AND #8 AND #14 AND #20
